# Spatial and temporal frequency band changes during infarct induction, infarct progression, and spreading depolarizations in the gyrencephalic brain

**DOI:** 10.3389/fnins.2022.1025967

**Published:** 2022-12-05

**Authors:** Modar Kentar, Roberto Díaz-Peregrino, Carlos Trenado, Renán Sánchez-Porras, Daniel San-Juan, F. Leonardo Ramírez-Cuapio, Niklas Holzwarth, Lena Maier-Hein, Johannes Woitzik, Edgar Santos

**Affiliations:** ^1^Department of Neurosurgery, University Hospital Heidelberg, Ruprecht-Karls-University Heidelberg, Heidelberg, Germany; ^2^Institute of Clinical Neuroscience and Medical Psychology, Medical Faculty, Heinrich Heine University, Düsseldorf, Germany; ^3^Department of Neurosurgery, Evangelisches Krankenhaus, Carl-von-Ossietzky University, Oldenburg, Germany; ^4^Epilepsy Clinic, National Institute of Neurology and Neurosurgery, Manuel Velasco Suárez, Mexico City, Mexico; ^5^Division of Intelligent Medical Systems, German Cancer Research Center, Heidelberg, Germany

**Keywords:** spreading depolarization, stroke progression, ECoG recording, power spectrum of signal decomposition, frequency bands

## Abstract

**Aim:**

To describe the spatial and temporal electrocorticographic (ECoG) changes after middle cerebral artery occlusion (MCAo), including those caused by spreading depolarization (SD) in the pig brain.

**Methods:**

The left middle cerebral arteries (MCAs) were clipped in six pigs. The clipping procedure lasted between 8 and 12 min, achieving a permanent occlusion (MCAo). Five-contact ECoG stripes were placed bilaterally over the frontoparietal cortices corresponding to the irrigation territory of the MCA and anterior cerebral artery (ACA). ECoG recordings were performed around 24 h: 1 h before and 23 h after the MCAo, and SDs were quantified. Five-minute ECoG signal segments were sampled before, 5 min, and 4, 8, and 12 h after cerebral artery occlusion and before, during, and after the negative direct current shift of the SDs. The power spectrum of the signals was decomposed into delta, theta, alpha, beta, and gamma bands. Descriptive statistics, Wilcoxon matched-pairs signed-rank tests, and Friedman tests were performed.

**Results:**

Electrodes close to the MCAo showed instant decay in all frequency bands and SD onset during the first 5 h. Electrodes far from the MCAo exhibited immediate loss of fast frequencies and progressive decline of slow frequencies with an increased SD incidence between 6 and 14 h. After 8 h, the ACA electrode reported a secondary reduction of all frequency bands except gamma and high SD incidence within 12–17 h. During the SD, all electrodes showed a decline in all frequency bands. After SD passage, frequency band recovery was impaired only in MCA electrodes.

**Conclusion:**

ECoG can identify infarct progression and secondary brain injury. Severe disturbances in all the frequency bands are generated in the cortices where the SDs are passing by.

## Introduction

Spreading depolarization (SD) is recognized as a negative direct current (DC) shift in the brain frequency range of <0.05 Hz, which propagates sequentially at adjacent recording sites. It is induced by severe neurological disorders, such as stroke, aneurysmal subarachnoid hemorrhage, intracerebral hemorrhage, traumatic brain injury, or epilepsy. SD produces a decrease in amplitudes of spontaneous activity, also known as spreading depression, in electrically active brain tissue (Hartings et al., 2011; Dreier et al., 2017). SDs have heterogeneous propagation patterns (Santos et al., 2017), making it difficult to detect SD using electroencephalography (EEG). The SDs contribute to the worsening of the penumbra and the infarct progression after an ischemic stroke (Ayata and Lauritzen, 2015). However, the specific anatomical and temporal frequency band disturbances produced by SDs are not well known.

**FIGURE 1:**
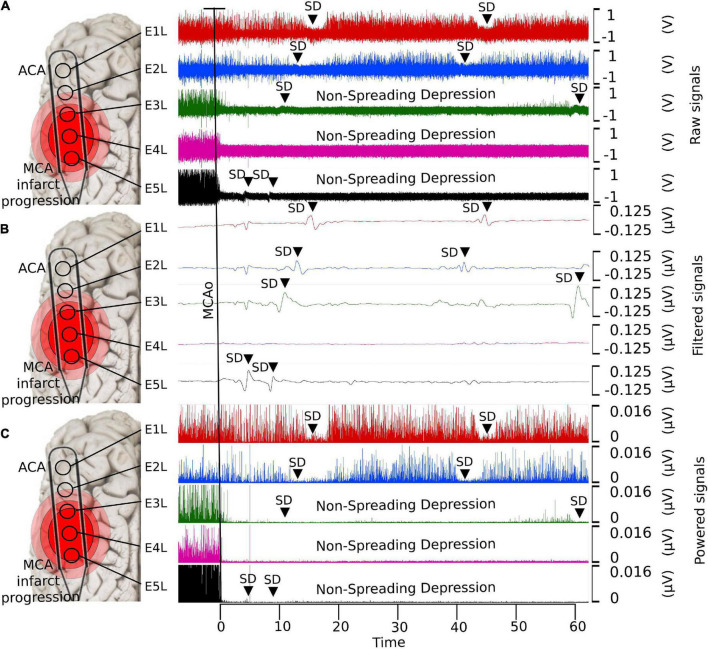
Detection of SDs using ECoG with different filters. **(A)** Raw data, **(B)** low-pass filtered, and **(C)** powered signal. With the low-pass filter, it is possible to observe the shape of the near-DC shift typic of the SDs. The non-spreading depression occurring after the MCAo is highlighted in the last two electrodes (E4L-E5L) at the raw and powered signal filters, which correspond to the site close to the MCAo. SD, spreading depolarization; ECoG, electrocorticography; DC, direct current; MCAo, middle cerebral artery occlusion.

During artery occlusion in the brain, there is a decrease in the spectral power of the frequency bands referred to as the non-spreading depression of activity (Figure 1; Dreier et al., 2017). How it occurs and behaves over time in humans is difficult to document because of the lack of electrocorticography (ECoG) monitoring during acute ischemic stroke. Moreover, dynamic pathophysiological changes in several brain areas after the spreading depression of spontaneous electrical activity should be investigated.

Similarly, scientists have studied infarct progression after stroke in humans using EEG and magnetoencephalography. The most consistent findings were the reduction of fast frequency bands and the predominance of delta and theta frequencies, which predict an unfavorable prognosis (Cillessen et al., 1994; Tecchio et al., 2005, 2007; Burghaus et al., 2007; Diedler et al., 2009; Moeller et al., 2011).

Due to the great complexity and variation in brain ischemia-induced pathophysiology, a consensus regarding the modifications of frequency bands after stroke is difficult to reach, except that brain activity appears to correlate with cerebral blood flow, oxygen, and glucose levels (Rabiller et al., 2015). Power declines in alpha, beta, and gamma frequencies are observed when the cerebral oxygen metabolism is critically reduced (Nagata et al., 1989). In addition, delta and theta rhythms seem to be reliable parameters correlating with cerebral blood flow and metabolic changes during focal ischemia in the cortex (Rabiller et al., 2015). Additionally, a lesion in the white matter induces irregular delta activity in the cortex overlying the infarct (Gloor et al., 1977; Rabiller et al., 2015).

The study of frequency band dynamics will help identify the presumed penumbra and infarct progression over time. Analysis of the initially unaffected areas facilitated the identification of the gradual changes produced by ischemia. Furthermore, the brain frequency bands will elucidate how harmful the SDs are and how they cause secondary damage to the penumbra and healthy structures.

Therefore, the primary aims of this study were to characterize the temporal and spatial changes in frequency bands 5 min and 4, 8, and 12 h after middle cerebral artery occlusion (MCAo) and during SD development.

## Materials and methods

### Experimental set-up

The Institutional Animal Care and Use Committee in Karlsruhe, Baden-Württemberg, Germany authorized the experimental protocol (Protocol No. G-13/15, G-148-15, G-69/16). The experiments were conducted in compliance with the University of Heidelberg Animal Ethics Policy of the Interfacultary Biomedical Faculty (IBF 347). The Animal Research: Reporting *In Vivo* Experiments (ARRIVE) guidelines were followed.

Ten female Landrace pigs (3–4 months old and 28–32 kg) were kept under general anesthesia using midazolam (2–10 mg/mL), propofol (1 mL/20 mg), and isoflurane (1–1.5%). Four animals were used to establish models and settings. Six animals were used for this study. The sample rate of all ECoG recordings was ≥200 Hz. Two subdural five-contact, platinum wall strip electrodes were used (Ad-tech, Racine, Wisconsin, USA). A ground electrode was placed in the zygomatic bone. The recording was performed using a Powerlab 16/SP analog-to-digital converter (ADInstruments, Sydney, Australia), which has 16 independent single-ended analog inputs with the signal referenced to the ground. The alternating current (AC) recorder had a 0.1-Hz filter. A notch filter (50 Hz) was applied to eliminate line disturbances. Registration and analysis of ECoG were performed using LabChart v7 (ADInstruments). After the experiment, animals were euthanized with intravenous potassium chloride under general anesthesia.

### Study design

A transorbital approach was used to present the left middle cerebral arteries (MCAs), as described previously (Schöll et al., 2017; Kentar et al., 2020). Up to four MCAs were found and occluded with aneurysm clips. For ECoG recording, extensive craniectomy and dura mater excision were performed over the temporal line of the swine to expose parts of the frontal and parietal lobes of both hemispheres. Five-contact ECoG strips were placed bilaterally on the cortex surface, corresponding to the MCA and anterior cerebral artery (ACA) territories. The distance between electrodes was 10 mm. ECoG signals from 10 electrodes were obtained: five electrodes (E1R–E5R) from the healthy right hemisphere and five electrodes from the insulted left hemisphere (E1L–E5L). E1 was located rostrally in the frontal hemisphere corresponding to the ACA territory, whereas E5 was caudal and coincided with the MCA territory. The location of the electrodes was verified by identifying the non-spreading depression of electrical activity in the caudal electrodes E4L and E5L (Figure 1). The durations of the ECoG recordings were planned to be 24 h; 1 h before MCAo, and 23 h after MCAo.

### Frequency analysis

**FIGURE 2:**
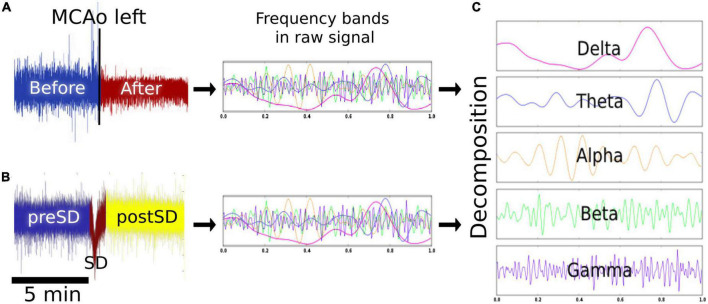
Signal analysis. **(A)** Effects of MCAo on the brain electrical activity. Five-minute epochs were obtained before, 5 min, and 4, 8, and 12 h after the left MCAo to analyze the modifications in the spectral power of each frequency band over time. **(B)** Effects of SD on the brain electrical activity. The SD signal was studied 5 min before (preSD), during (SD), and 5 min after the near DC-shift (postSD) to observe the alterations caused by the development and passage of the SDs. **(C)** Signal decomposition: the signal segments were decomposed to delta, theta, alpha, beta, and gamma bands. MCAo, middle cerebral artery occlusion; SD, spreading depolarization; DC, direct current.

To study infarct progression, 5-min ECoG signal segments were obtained 5 min before MCAo, as well as 5 min and 4, 8, and 12 after MCAo (Figure 2A). SD is presented as a negative near DC-shift (NDCS) and as a decrease in power in the ECoG bands in adjacent electrodes using an AC recorder (Dreier et al., 2017). For SD examination, 5-min signal segments before and after the NDCS of the SDs were analyzed. Moreover, the NDCS of the SDs were studied, presenting a mean duration of 51.3 s (σ = ±14.1 s). For reliable SD evaluation, the SDs included in the analysis had signal segments of 5 min before and after the NDCS free of artifacts and other SDs. The ECoG recording segments before, during, and after the NDCS were referred to as “preSD,” “SD,” and “postSD” (Figure 2B).

For signal decomposition, the discrete 512-point Fourier transform (Hanning window) was computed for each data segment and, subsequently, the power spectrum (Al-Fahoum and Al-Fraihat, 2014; Delimayanti et al., 2020). For each segment, we estimated the mean power spectrum corresponding to each frequency band: delta (0.1–4 Hz), theta (4–7 Hz), alpha (8–12 Hz), beta (13–31 Hz), and gamma (32–45 Hz). The power calculation was performed using customized MATLAB programs (MathWorks, Natick, MA) (Figure 2C). To determine infarct progression, the spectral power of the frequency bands was calculated every 4 h starting from the MCAo, with four timepoints plus the baseline obtained 5 min before the MCAo. For each pig (*n* = 6), the power spectrum of each frequency band was calculated for each timepoint to obtain a dataset of six elements for each timepoint. Similarly, the SDs were collected and analyzed in periods of 3 h per electrode, with eight time lapses in total. The power spectrum of each frequency band was computed for each SD phase (preSD, SD, and postSD), resulting in a dataset of eight elements for each phase. To observe the results in the right hemisphere, please see Supplementary Figure 1.

### Statistical analysis

SPSS v25 (IBM, Armonk, NY) was used for statistical analysis and plots were created using GraphPad Prism 8.0.1 (GraphPad Software, San Diego, CA). Data were subjected to Shapiro–Wilk analysis to determine their distribution. The data had a non-normal distribution. Therefore, non-parametric tests were conducted, as follows:

(A) Spectral power of the frequency bands after MCAo: The Wilcoxon matched-pairs signed-rank test was applied to determine the variations over time between the spectral powers of the frequency bands. For comparison, the frequency bands gathered before MCAo were used as a reference to compare the power alterations of the frequency bands 5 min immediately after artery occlusion (0 h) and at 4, 8, and 12 h.
(B) Spectral power of the frequency bands during and after SDs: The five frequency bands within 5 min of the preSD period were used as the baseline to evaluate the power changes at SD and postSD, respectively. For the comparison between preSD and SD and between preSD and postSD, the Wilcoxon matched-pairs signed-rank test was executed.

## Results

**FIGURE 3:**
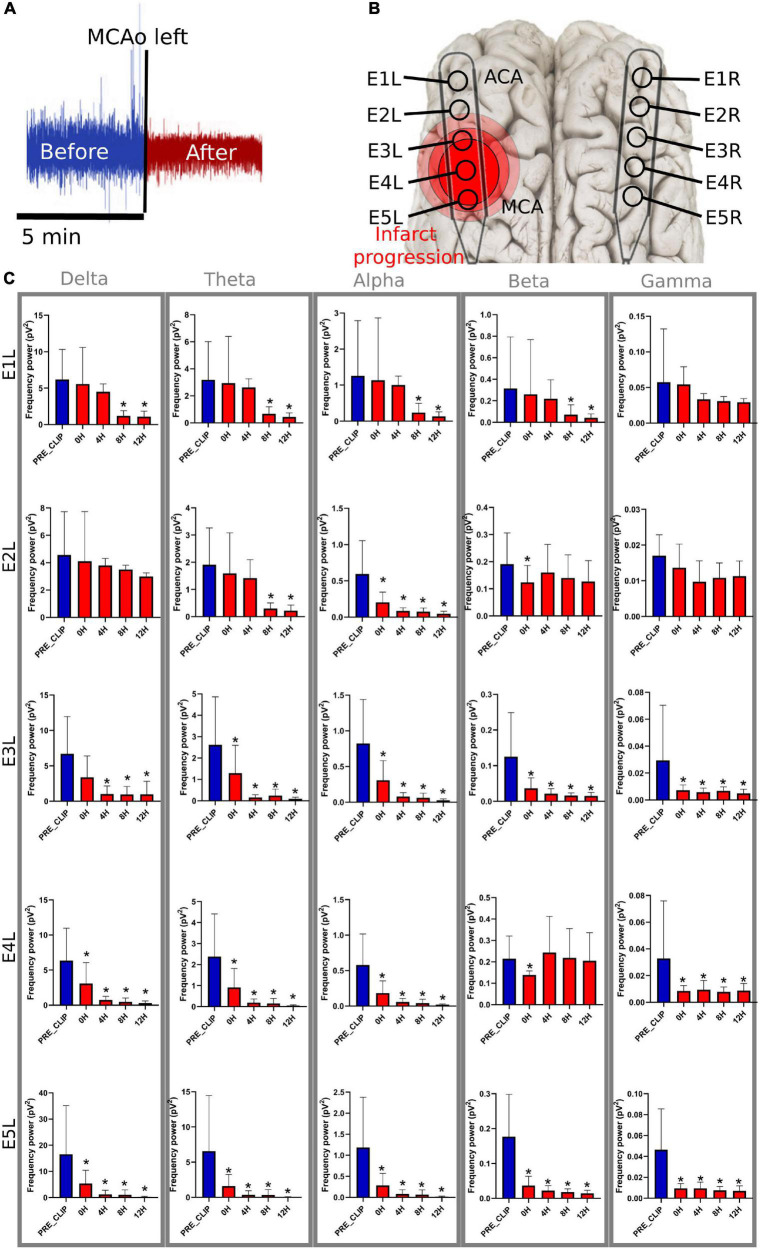
Frequency bands in the left electrodes after MCAo. **(A)** The 5-min signal segment before MCAo was used as the baseline to compare the changes in the spectral power at 0 h (5 min immediately after), 4, 8, and 12 h after MCAo. For each timepoint, 5 min of the ECoG recording were used for the analysis. **(B)** The electrodes were placed parallel over the primary frontoparietal cortex. Electrodes E1L-E5L acquired information from the left ischemic hemisphere. It is speculated that E5 was set over the cerebral cortex near the MCAo, and E1 over the ACA territory. **(C)** The significant drop in the spectral power of the frequency bands is represented as * (≤0.05). MCAo, middle cerebral artery occlusion; ECoG, electrocorticography; ACA, anterior cerebral artery; pV2, squared picovolts.

**FIGURE 4:**
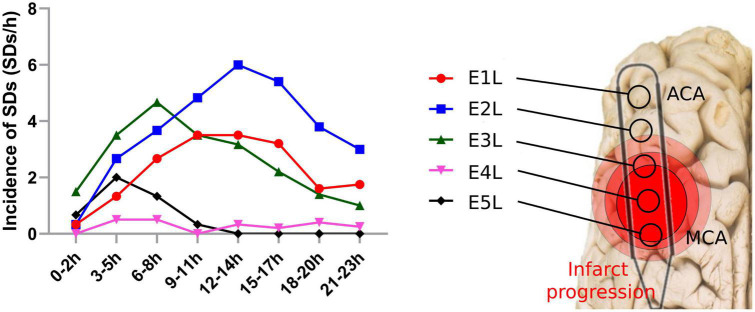
Spreading depolarization incidence after MCAo in each electrode. SDs were quantified in each electrode of the affected hemisphere after the MCAo. SD, spreading depolarization; MCAo, middle cerebral artery occlusion.

**FIGURE 5:**
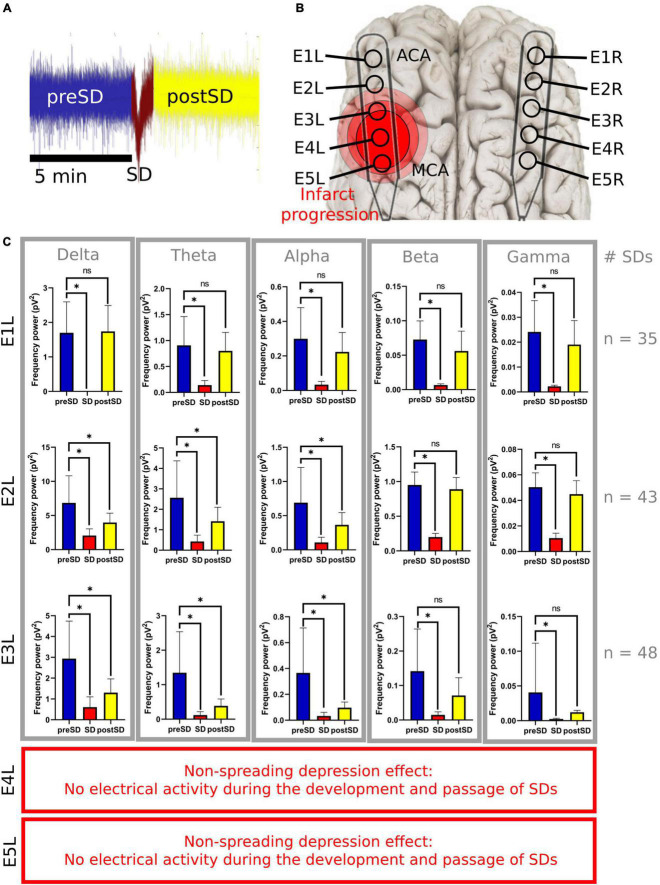
Frequency bands in the left electrodes during and after SDs. **(A)** The 5-min signal segment before the SD (preSD) was used as the baseline to compare the variations in the spectral power during (SD) and after the SD (postSD) segments. Five minutes of the ECoG recording were used to analyze postSD. Meanwhile, the mean recording time of the near DC-shift of the SD was 51.3 s (σ = ±14.1s). **(B)** E1L was in the ACA territory and E5L was closer to the MCA territory. **(C)** Statistical analysis of SD segments: preSD was used as the baseline to compare the variations in the spectral power at SD and postSD segments, respectively. *Statistically significant difference among SD segments (*p* ≤ 0.05). E4L and E5L recorded isoelectric SDs with few or null electrical activities not relevant for the spectral power analysis of the frequency bands. SD, spreading depolarization; ECoG, electrocorticography; DC, direct current; ACA, anterior cerebral artery; MCA, middle cerebral artery; pV^2^, squared picovolts.

Four experiments lasted 24 h, one 18 h, and one 13 h; the latter experiments were shorter due to technical problems during the ECoG recording. After MCAo, the following findings were elucidated: (1) the electrode over the frontal cortex supplied by the ACA (E1L) did not undergo changes in the frequency bands during the first 8 h, while electrodes over the parietal cortex close to the MCAo (E5L and E4L) showed a decrease in spectral power in all frequency bands immediately after MCAo, which was sustained for the rest of the recording (Figure 3); (2) specific anatomical and temporal patterns of power reduction were observed at different frequencies according to the location of the electrodes (Figure 3); (3) the peak of the highest incidence of SDs differed depending on the anatomic location of the electrodes (Figure 4); (4) during the SD period, all frequency bands were suppressed in both the ACA (E1L) and MCA (E2L and E3L) (Figure 5); (5) E2L and E3L showed impaired recovery in postSD in all the frequency bands when the penumbra was suspected, whereas the E1L preserved recovery of all frequency bands in the same period, which might be classified as an unaffected zone (Figure 5); and (6) the right healthy hemisphere showed no significant ECoG changes over the whole experiment (Supplementary Figure 1).

### Spectral power of the frequency bands after middle cerebral artery occlusion

Spectral power analysis was performed for only 12 h to maximize the reliability of the results because two animals did not complete the 24 h for technical problems (mainly artifacts that altered the ECoG signal).

E1L, corresponding to the ACA territory, did not show any modifications in the spectral power of the frequency bands during the first 8 h of blood restriction. However, 8 h later, the power of the delta, theta, alpha, and beta frequencies diminished significantly, maintaining intact gamma power over time, showing a pattern of delayed damage.

E2L suffered soon after MCAo, with an alpha power drop and a temporary reduction in beta power. The theta power frequency decreased after 8 h. Delta and gamma frequencies were preserved in the electrode during the experiment. E3L, the power of all brain frequencies except delta, decreased immediately after the MCAo. Second, the delta band suffered a delayed drop after 8 h of blood flow constraints.

E4L reported a spectral power decline in all frequency bands; nevertheless, the beta band recovered 4 h later. E5L registered an initial decrease in all frequencies without recovery over time (Supplementary Table 2 and Figure 3).

### Incidence of spreading depolarizations after middle cerebral artery occlusion

In the left ischemic hemisphere, the SDs showed a peak incidence at the electrodes over the cortex supplied by the MCA and ACA. E1L, located over the ACA territory, recorded 99 SDs, with the highest incidence between 12 and 17 h after MCAo. Similarly, E2L displayed the highest SD incidence, recording 163 SDs and exhibiting a peak between 12 and14 h. E3L detected 120 SDs and showed a crest at 6–8 h. E4L recorded 12 SDs, registering an incidence peak at 3–8 h, whereas E5L showed 26 SDs with a peak at 3–5 h, and later signs of brain electrical depression (Figure 4).

### Spectral power of the frequency bands during and after the spreading depolarization

To facilitate the decomposition and analysis of the SDs, only SDs with signal segments of 5 min before and after the DC shift, without electrographic artifacts and other SDs, were studied. There were 35, 43, and 48 SDs included in the E1L, E2L, and E3L groups, respectively. No SDs from E4L and E5L were included because of the low or null brain activity found early in the ECoG recording after MCAo (Figure 1).

The SD and postSD periods were compared to the preSD segment. E1L, E2L, and E3L showed a significant spectral power drop in all frequency bands during SD. Furthermore, the spectral power of all the frequency bands remained reduced in postSD only in E2L and E3L. No differences were found in the frequency bands at postSD in E1L (Supplementary Table 3 and Figure 5). Thus, the brain cortex underlying E1L recovered its spectral power to basal levels shown in the preSD, but the gray matter at both E2L and E3L withheld the damage caused by SD passage.

## Discussion

ECoG recordings were helpful in detecting and characterizing modifications in frequency bands caused by MCAo and SDs during the onset and evolution of cerebral infarction in gyrencephalic brains. We could discern which zones were affected by ischemia and SDs, which frequency bands were disrupted, by how much, and how they evolved over time. Thus, ECoG might be implemented as a bedside device to guide treatment approaches, such as decompressive craniectomy or neuroprotective measurements, to counter the deleterious effects of brain ischemia and avoid secondary injury.

Variations in the spectral power of frequency bands are difficult to document in patients with acute ischemic stroke because they occur seconds to minutes after occlusion, where non-invasive and invasive EEG recordings are usually not available and are not routinely used for diagnosis or treatment. Experiments in pigs overcome limitations in small laboratory animals such as rodents, as pigs allow for more space to set the electrodes, more precision in locating the anatomic sources, and their brains are more similar to the human brain (gyrencephalic), thus allowing the use of the same measurement instruments as in humans.

In contrast, the main limitations of EEG are related to the tissue barrier of the scalp, which prevents the detection of low-energy brain activity, such as frequencies higher than 100 Hz and those lower than 0.1 Hz (Rabiller et al., 2015) and low spatial resolution when detecting cerebral ischemia (Faught, 1993). In these experiments, it was possible to monitor the exact moment of infarction and its evolution across the brain cortex, recognizing the pathophysiological phases that influence the outcome of patients.

The anesthetic effect on brain activity in humans and pigs should be considered during the evaluation of the frequency bands in cerebral ischemic scenarios. The anesthetic agents had a dynamic effect on cerebral activity during the different phases of induction, maintenance, and emergence. The anesthetic drugs have specific anatomic regions of action and the frontoparietal cortex is one of their targets, the same place the electrodes were set. It was expected that the anesthetic agents such as midazolam, propofol, and isoflurane induce an unconscious state by modulating the brain activity of swine when attenuating the fast frequencies and increasing the slower ones (Akeson et al., 1993; Mäkiranta et al., 2002; Bojak et al., 2015; Mirra et al., 2022). Furthermore, isoflurane is capable to suppress SD development. Thus, the SD accounts might be decreased in all the experiments (Takagaki et al., 2014; Klass et al., 2018).

It was observed that the electrodes in the right hemisphere reported a lowering in the spectral power in all the frequency bands compared to the left electrodes but accentuated in beta and gamma bands (Supplementary Figure 1 and Supplementary Table 1). Nonetheless, the baseline power spectrum of the frequency bands at the right hemisphere remained stable over time, even when the left side suffered an ischemic insult.

Three conditions might influence the basal spectral power of the frequency bands since the beginning of the experiment: (I) The anatomical differences among the hemispheres; (II) The asymmetric activity of the brain activity; (III) The heterogeneous effect of the anesthesia in the cortex.

Anatomical variations are found in the frontoparietal cortices, being the right side more extensive than the left one. As a result, the location of the electrodes is not completely symmetric between hemispheres, and the gathered signals and frequency bands might slightly vary (Toga and Thompson, 2003; Goldberg et al., 2013). Additionally, the physiological interhemispheric asymmetry in brain activity is exemplified in the left hemisphere, which exhibits a high dominance of fast frequencies in the resting state than the right one (Mahjoory et al., 2020; Mason et al., 2022). Regarding anesthesia, it has a heterogeneous and dynamic effect across the brain cortex, provoking deep alterations in the cerebral metabolic rate, the brain blood oxygenation, and the cerebral blood flow in some specific brain areas. In our experiments, the right hemisphere may be the most affected by the anesthetic agents (Ciobanu et al., 2012; Hudetz, 2012; Bojak et al., 2015; Halder et al., 2021).

### Spectral power of the frequency bands after clipping the middle cerebral artery

Immediately after artery clipping, a spectral power weakening of the frequency bands at various scales was observed in some electrodes. This change is defined as a non-spreading depression of electrical activity according to nomenclature standards (Dreier et al., 2017), which we previously reported (Sánchez-Porras et al., 2022). When cerebral blood flow reaches 12 mL/100 g/min or less, infarction becomes evident because of the progressive loss of transmembrane potential gradients of neurons (Rabiller et al., 2015). In E4L and E5L over the parietal cortex, the cerebral blood flow values surpassed this infarct threshold, showing a non-spreading depression of spontaneous brain activity in all frequency bands that lasted the whole experiment (Figure 1).

If cerebral blood flow is below the ischemic threshold but maintained above the infarction threshold, the effect on metabolism or cell survival is still reversible, with visible electrical activity in the delta frequency, as seen in E2L and E3L in the first 4 h after stroke. Delta band is well-recognized as prognostic markers in stroke patients (Schaul et al., 1986) and are predictive of a malignant course (Burghaus et al., 2007). Even at later stages (from 4 to 10 days), it has a negative prognostic prediction (Assenza et al., 2013). Speculative cerebral blood flow at E2L–E3L may reach levels of 20–30 mL/100 g/min, where the abnormal release of glutamate occurs (Sharbrough et al., 1973; Hossmann, 1994; Guyot et al., 2001; Gallinat et al., 2006; Rabiller et al., 2015).

Additionally, the maintenance of fast frequencies, mainly the gamma band, was present in the electrodes distant to the MCAo (E1L and E2L) and absent in those located close to vessel clipping (E4L and E5L), indicating that the presence of this frequency band might a biomarker of the integrity of the neural network. It is not clear whether beta recovery occurred in E2L and E4L. Nonetheless, we expected to find a steady decay of the beta band mainly close to the MCAo at the E4L as a resemblance to neural circuitry dysfunction found in the adjacent electrodes E1L, E3L, and E5L and other studies (Cillessen et al., 1994; Tecchio et al., 2005, 2007; Burghaus et al., 2007; Diedler et al., 2009; Moeller et al., 2011; Hertle et al., 2016). The heterogeneous infarct evolution might have an influence on the preservation of the beta band at E2L and E4L.

After the characterization of the frequency bands in each electrode, it can be inferred that E1L represents the initial normal cortex with secondary injury, E2L and E3L represent the transitional cortex or penumbra, and E4L and E5L represent the ischemic core (Figure 3). Further imaging and neurophysiological studies are needed to corroborate these neurophysiological findings.

### Incidence of spreading depolarization

It is important to note that infarct progression is related to SD incidence. The infarct core zone was recorded by E4L and E5L, having fewer SDs, displaying the early appearance of SDs after MCAo but a premature cessation of SD development. The arrest of SD development indicates a lack of excitability in the neural network due to irreversible neuronal damage. For E1L, E2L, and E3L, the incidence of SDs was higher, with a steep peak in SD incidence at the late stages of the ECoG recording. Normal and salvageable tissue, which was recorded by E1L–E3L, would be the target for onset due to their preserved neural network and excitability. As a result, it is possible to differentiate between normal and damaged tissues based on their SD incidence. The benefit of recognizing these zones is the implementation of approaches to block SD development and test new strategies for neuroprotection.

One advantage of the swine model is that the periodicity of SD is more regular and the inter-event intervals are more prolonged in comparison to the lyssencephalic brain models, allowing a profound study of the SDs underpinning mechanisms.

### Spectral power of the frequency bands during and after spreading depolarization

All frequency bands suffered significant depression during SD passage in the analyzed electrodes (E1L, E2L, and E3L). A similar phenomenon was observed in electrodes located in the tissue with the most restrictive blood supply after MCAo (E4L and E5L), confirming the ischemic effect of SD in the penumbra and normal cortical zones (Sharbrough et al., 1973; Strong et al., 2006; Dreier et al., 2009; Ayata and Lauritzen, 2015; Østergaard et al., 2015). Thus, SDs reach sites far from their origin, causing distant ischemic effects (Santos et al., 2016). Using laser speckle analysis, it has been documented that SDs can move to the ACA territory, producing secondary cerebral blood flow arrest in a matter of minutes (Kentar et al., 2020; Sánchez-Porras et al., 2022).

However, we identified three differences between E1L and both E2L and E3L. First, E1L was not affected by MCAo in the early stages of the ECoG recording. Second, E1L registered fewer SDs than E2 or E3L in the whole experiment (Figure 4). Finally, there was a full recovery of the spectral power of all frequency bands at E1L, while all the frequency bands remained depressed at E2 and E3L during postSD (Figure 5). These three statements support the SD characterization according to the anatomical regions, confirming the location of the recorded zones: E2L and E3L were located in the penumbra and E1L in normal tissue. The disrupted recovery of all frequency bands in the E2L and E3L would result in prolonged depression of electrical activity. SDs might not be compensated by tissue because of neuronal death and net malfunction in these areas caused by infarction progression and SDs. These findings coincide with the results presented in the DISCHARGE-1 study, which showed that the total SD-induced depression duration of a recording better predicts delayed infarction than other variables (Dreier et al., 2022). In our work, the high SD and prolonged depression time of the frequency bands forecast the delayed evolution of the infarction.

SDs were also detected in E4L and E5L as isoelectric SDs (Figure 1), where low or no electrical activity was present. Thus, spectral power analysis would not provide any additional information to the two electrodes. As expected, clusters of agglomerated SDs were detected in the electrodes. A re-entrance phenomenon would probably help explain the high incidence of SDs in early ECoG recordings (Santos et al., 2014). Once the energy in this region is exhausted, few or no SDs appear in the ischemic core. Subsequently, a higher incidence occurred in the penumbra (E2L and E3L), reaching normal tissue in E1L.

### Effect of middle cerebral artery occlusion and spreading depolarizations in further cortical zones

During the study, no modifications in the spectral power of the frequency bands were observed in the right hemisphere, either during MCAo or during SD development or passage. Nevertheless, it is impossible to discard the possibility that some of the changes seen close to the ACA territory could be observed in other brain areas.

In previous studies, researchers reported alterations in frequency bands on the contralateral side. Interestingly, the distant cortices displayed the same brain signal pattern observed in the primary cortical injury: the suppression of fast frequencies and the maintenance and magnification of slower ones (Tecchio et al., 2005, 2007; Assenza et al., 2013).

### Study limitations

Our speculations about the core-penumbra map should be corroborated by studying the modifications in the cerebral blood flow and oxygen metabolism timewise with direct methods, such as single-photon emission computed tomography (SPECT), positron emission tomography (PET), magnetic resonance with contrast agents, and indirect technics like transcranial Doppler ultrasound imaging, phase-contrast MRI, and near-infrared spectroscopy (NIRS). The cytoarchitectonic changes must be assessed by histological analysis to determine the final infarct volume and verify the disposition of the core, penumbra, and healthy tissue. Finally, it is relevant to record remote brain areas to identify any distant injury caused by MCAo and SDs.

## Data availability statement

The datasets presented in this study can be found in online repositories. The names of the repository/repositories and accession number(s) can be found below: Diaz Peregrino, Roberto (2022), “SD_Frequency_Power”, Mendeley Data, V1, doi: 10.17632/gy8t7vwmtz.1 Diaz Peregrino, Roberto (2022), “Infarction_Progression_Frequency_Power”, Mendeley Data, V1, doi: 10.17632/rz3xyynvnc.1 Diaz Peregrino, Roberto (2022), “SD_Accounts”, Mendeley Data, V1, doi: 10.17632/x5k49h5x6m.1.

## Ethics statement

The animal study was reviewed and approved by the Institutional Animal Care and Use Committee in Karlsruhe, Baden-Württemberg, Germany.

## Author contributions

MK performed the experiments and wrote the manuscript. RD-P assisted in the experiments, performed the analysis, and wrote the manuscript. CT performed the analyses and wrote the manuscript. RS-P performed the experiments and corrected the manuscript. DS-J, FR-C, NH, LM-H, and JW corrected the manuscript and provided the scientific support. ES designed the project, performed the experiments, and wrote the manuscript. All authors contributed to the article and approved the submitted version.
