# Supplementary material for: Spatial and temporal frequency band changes during infarct induction, infarct progression, and spreading depolarizations in the gyrencephalic brain
Source: Front Neurosci. 2022 Dec 5;16:1025967. doi: 10.3389/fnins.2022.1025967 (PMC9769704; doi:10.3389/fnins.2022.1025967)
Supplement: Supplementary file 1 [file Data_Sheet_1.docx]

Supplementary Material

# Supplementary Figures and Tables

## Supplementary Figure


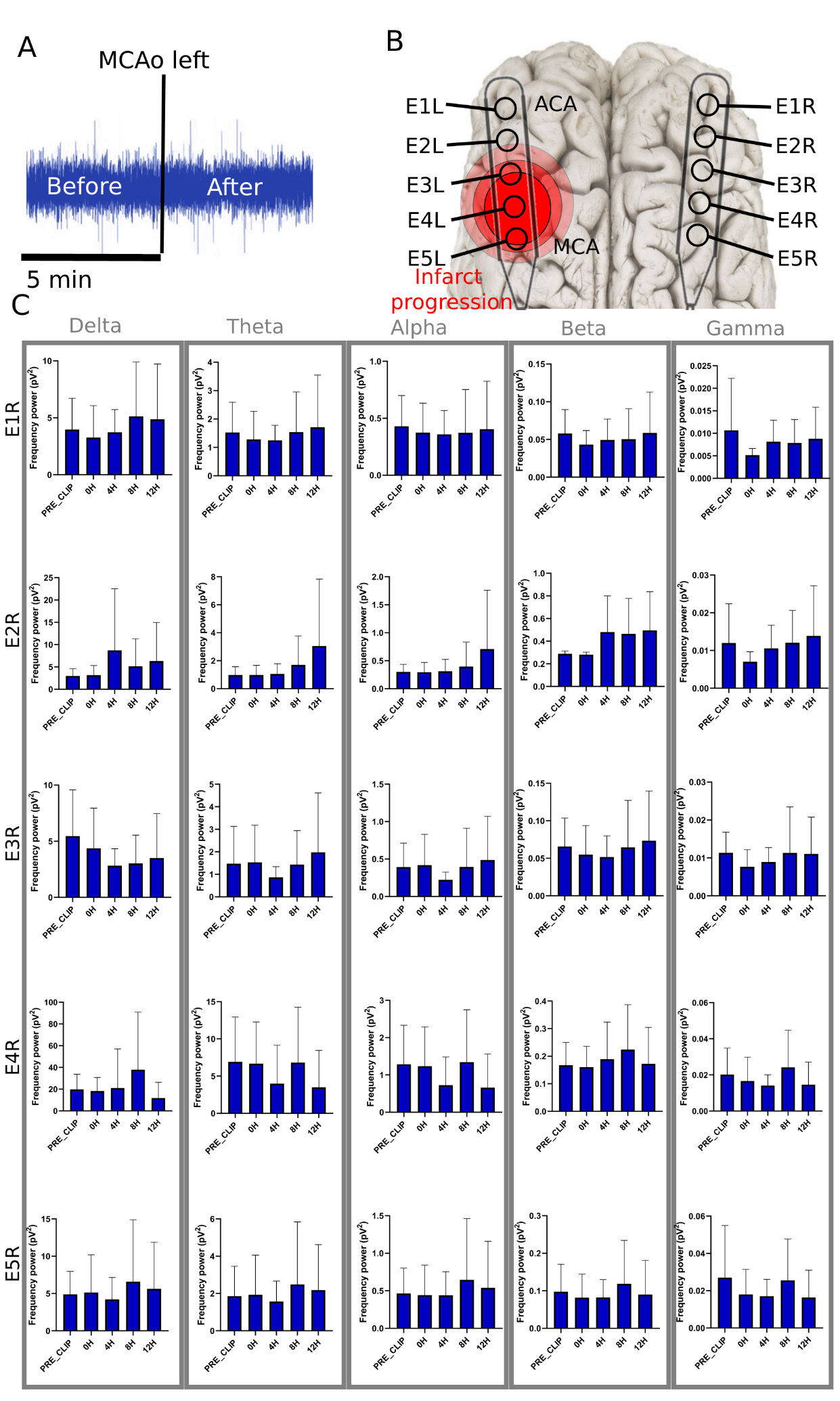


Supplementary Figure 1. Frequency power in the right electrodes after MCAo. (A) The 5-min signal segment before MCAo was used as the baseline to compare the changes in frequency power at 0 h (5 min immediately after), 4 h, 8 h, and 12 h after MCAo. For each timepoint, 5 min of the ECoG recording were used for the analysis. (B) The electrodes were placed parallel over the primary frontoparietal cortex. Electrodes E1R-E5R recorded the signal data from the right non-ischemic hemisphere. (C) The significant drop in power of the frequencies is represented as * (≤ 0.05). MCAo, middle cerebral artery occlusion; ECoG, electrocorticography; ACA, anterior cerebral artery; pV^2^, squared picovolts.

## Supplementary Tables
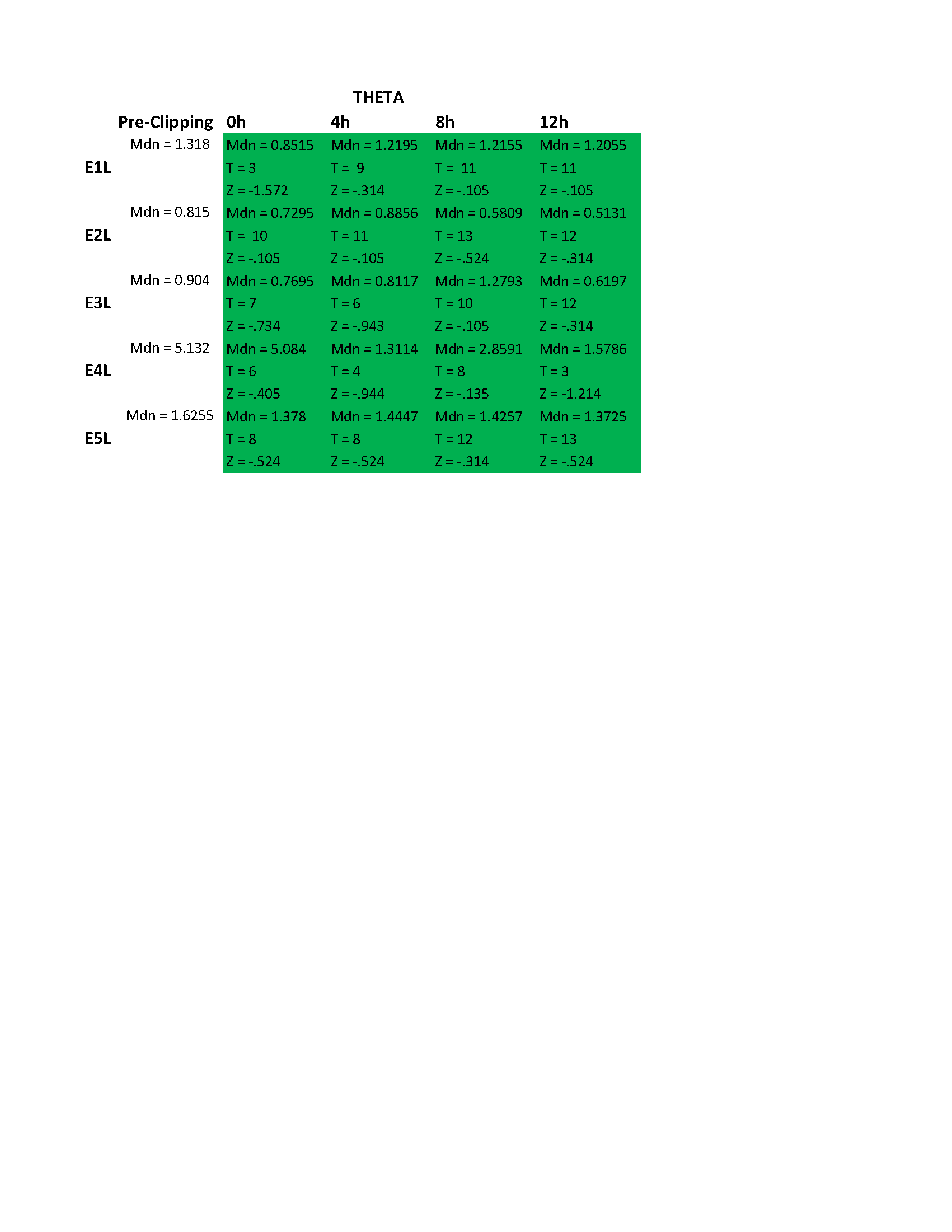

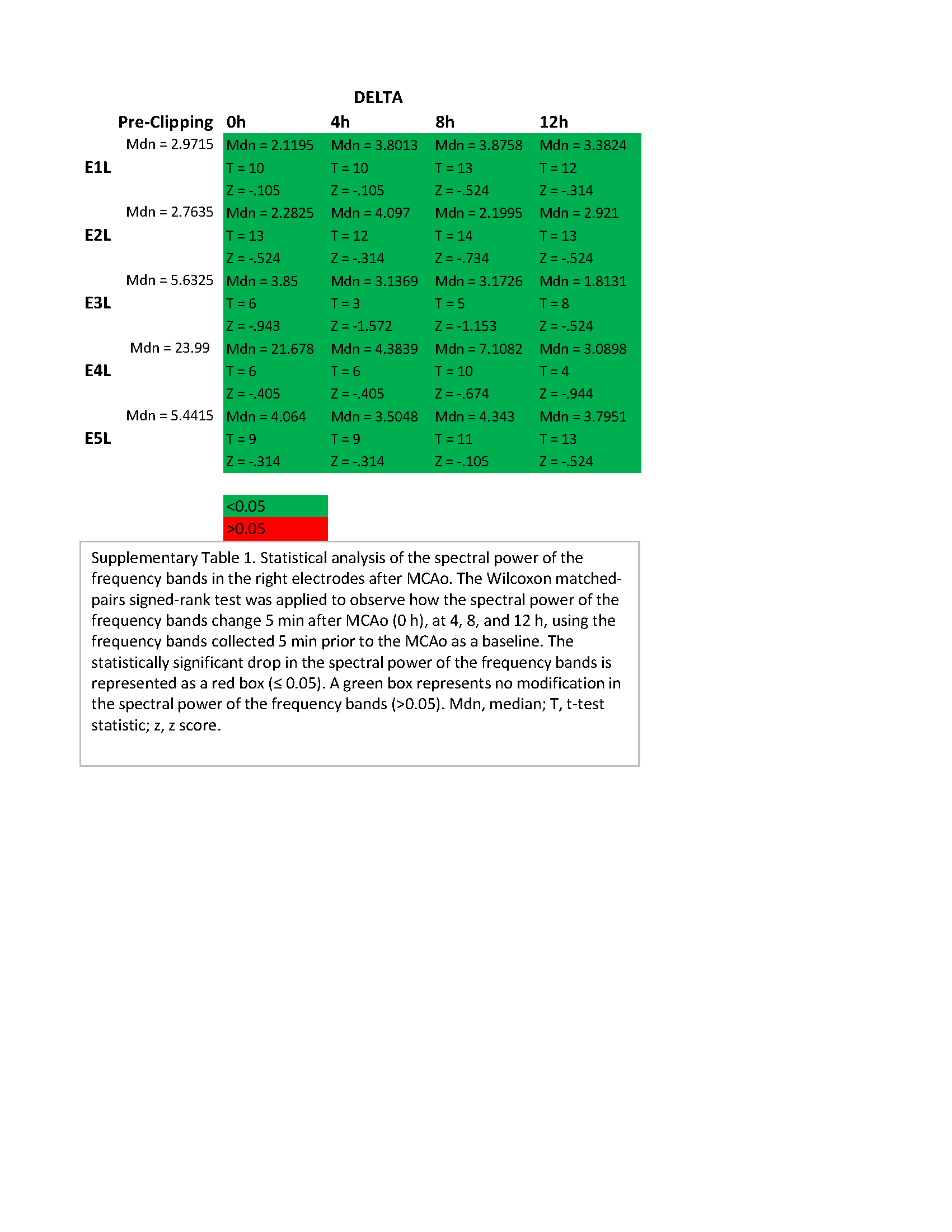


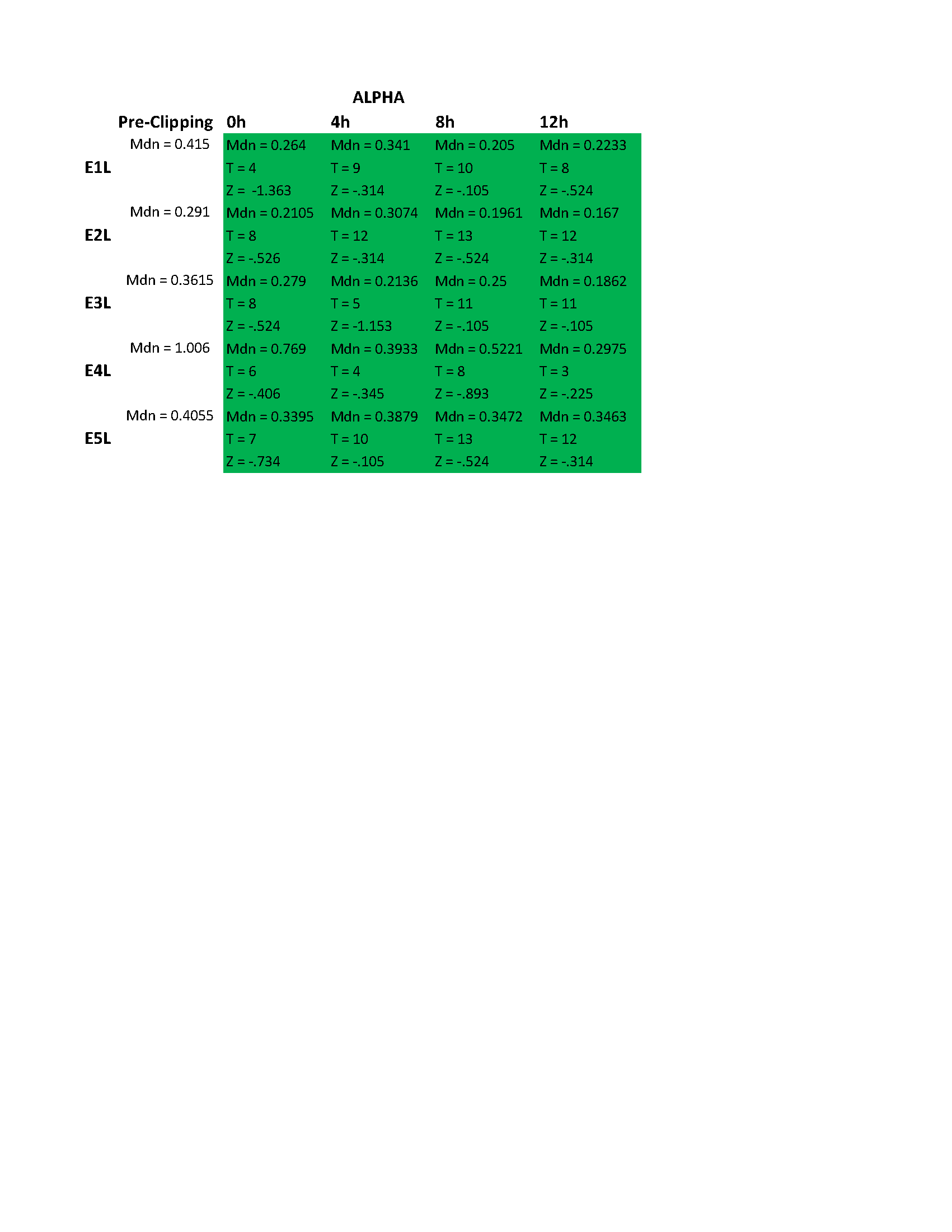

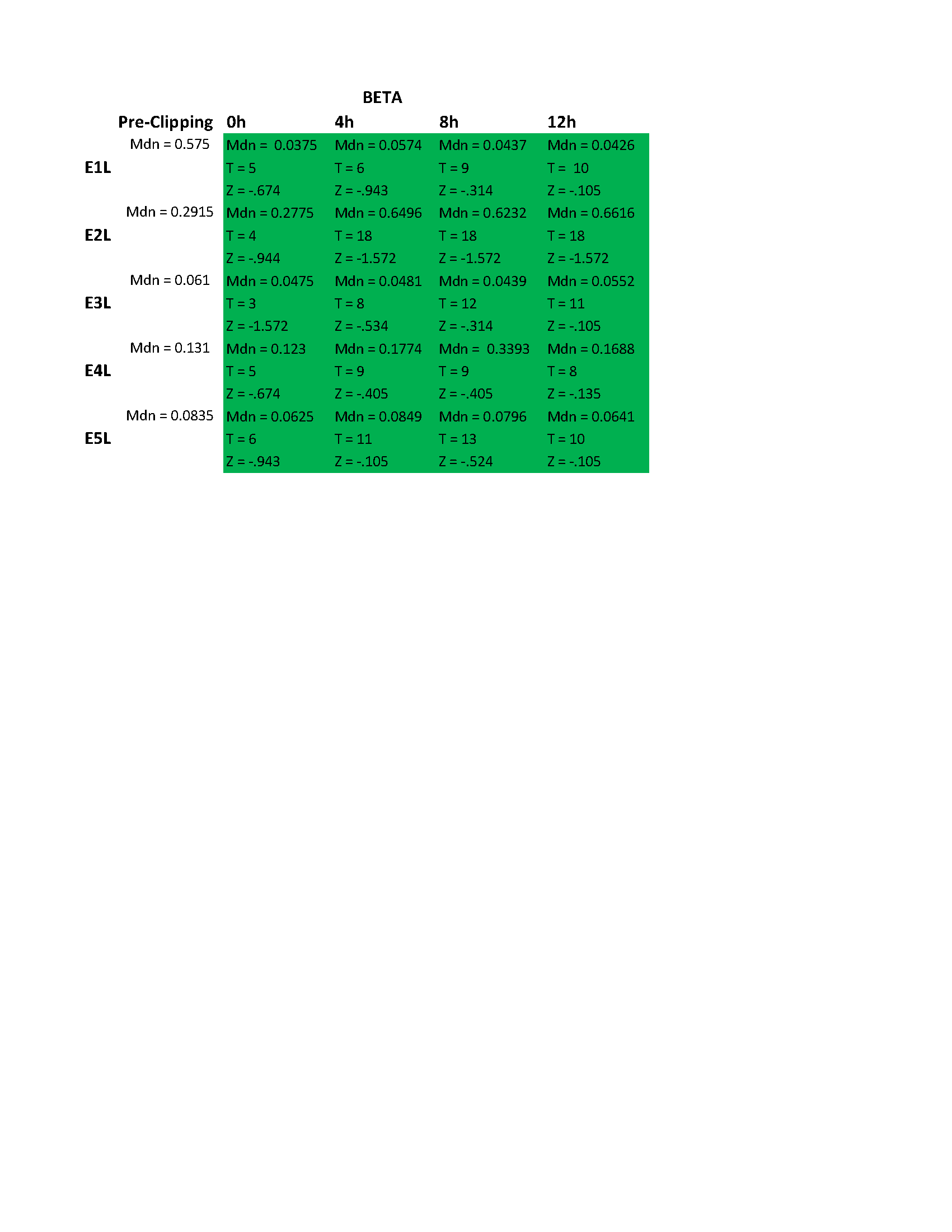


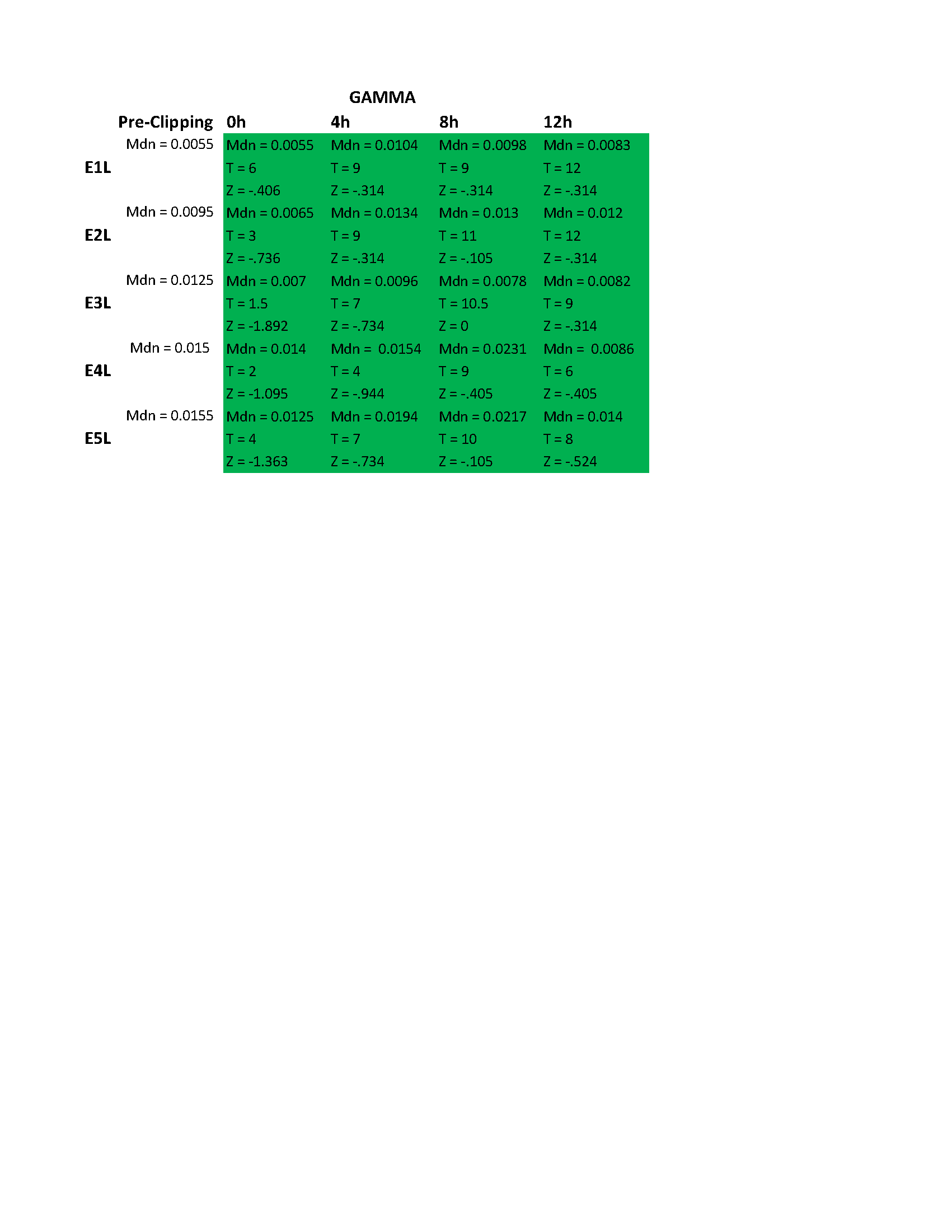


Supplementary Table 1. Statistical analysis of the spectral power of the frequency bands in the right electrodes after MCAo. The Wilcoxon matched-pairs signed-rank test was applied to observe how the spectral power of the frequency bands change 5 min after MCAo (0 h), at 4, 8, and 12 h, using the frequency bands collected 5 min prior to the MCAo as a baseline. The statistically significant drop in the spectral power of the frequency bands is represented as a red box (≤ 0.05). A green box represents no modification in the spectral power of the frequency bands (>0.05). Mdn, median; T, t-test statistic; z, z score.


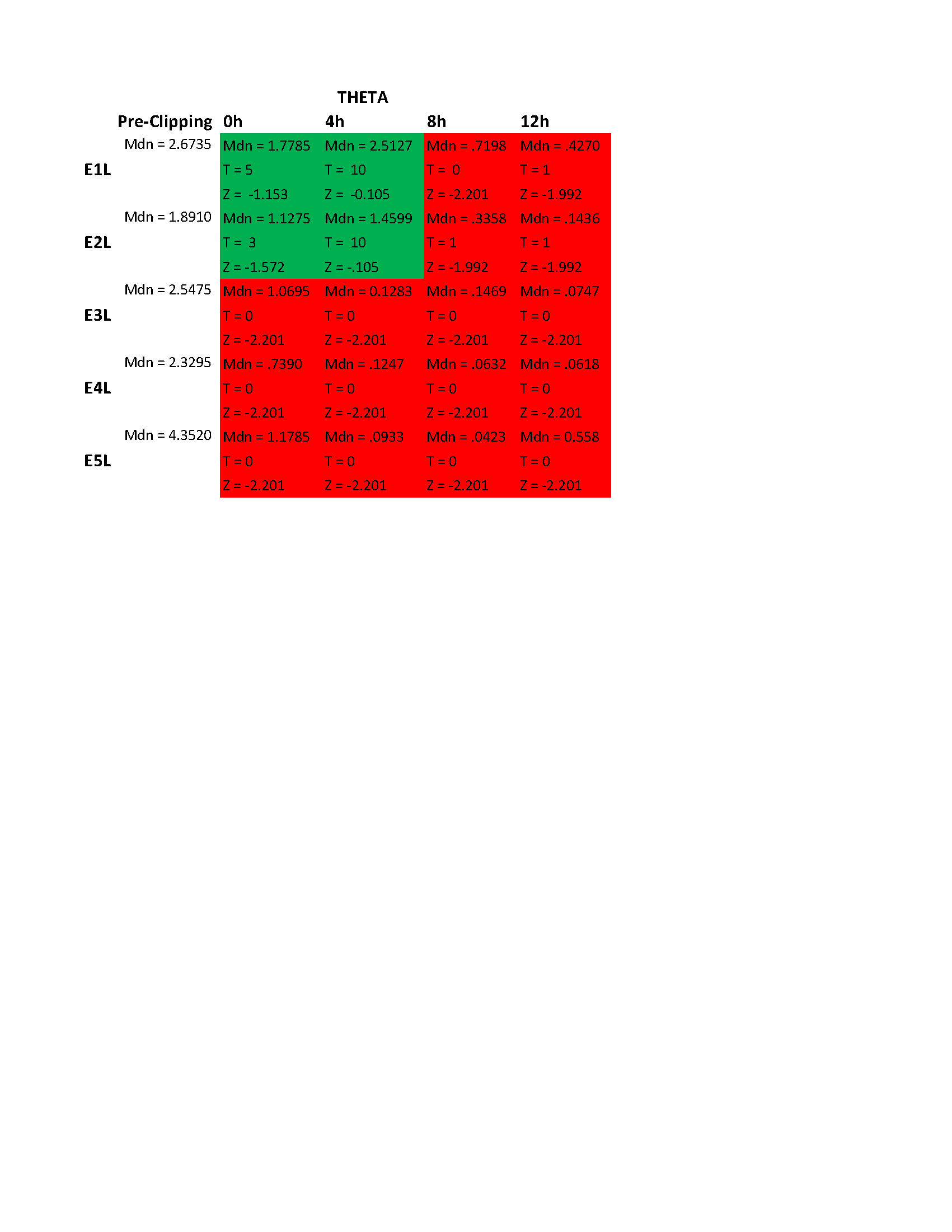

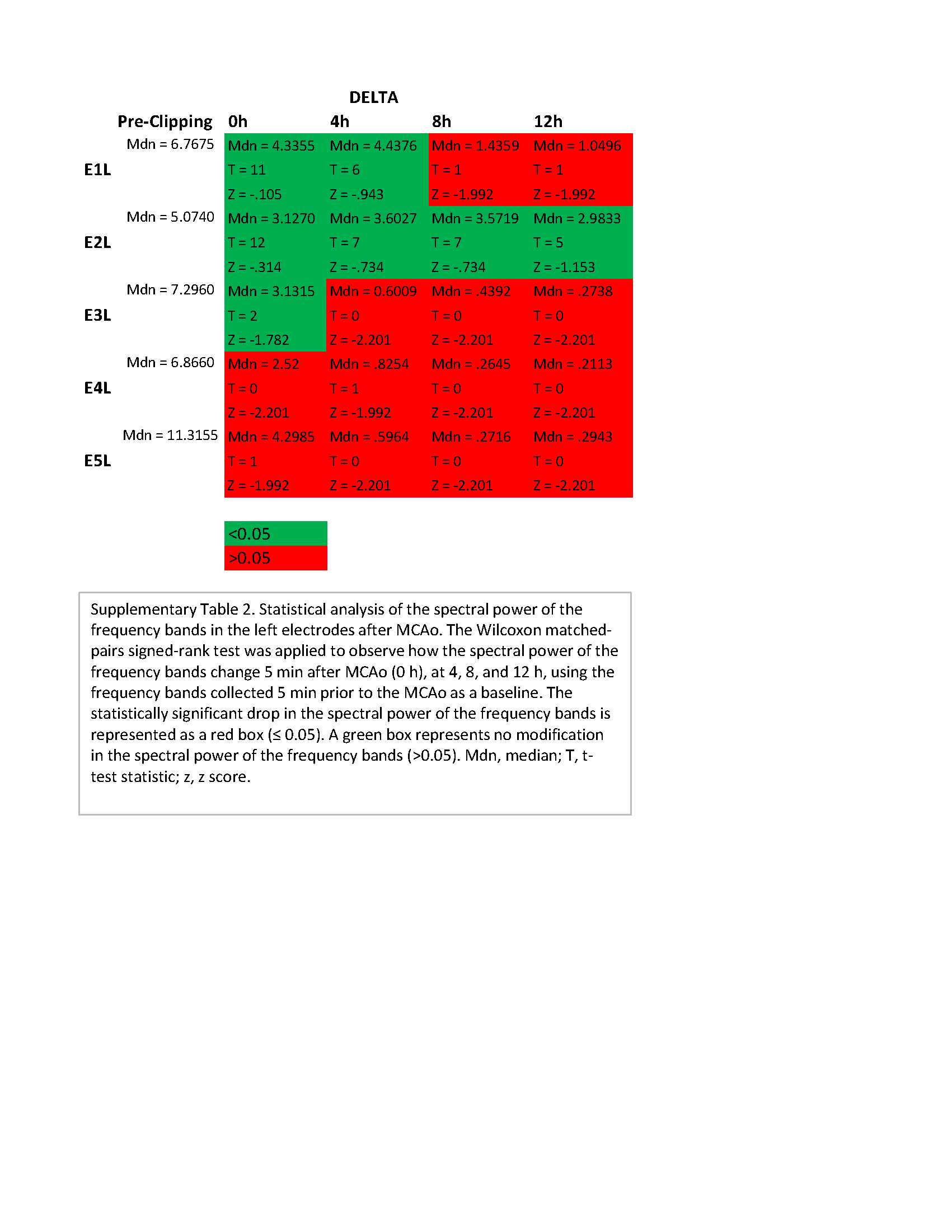


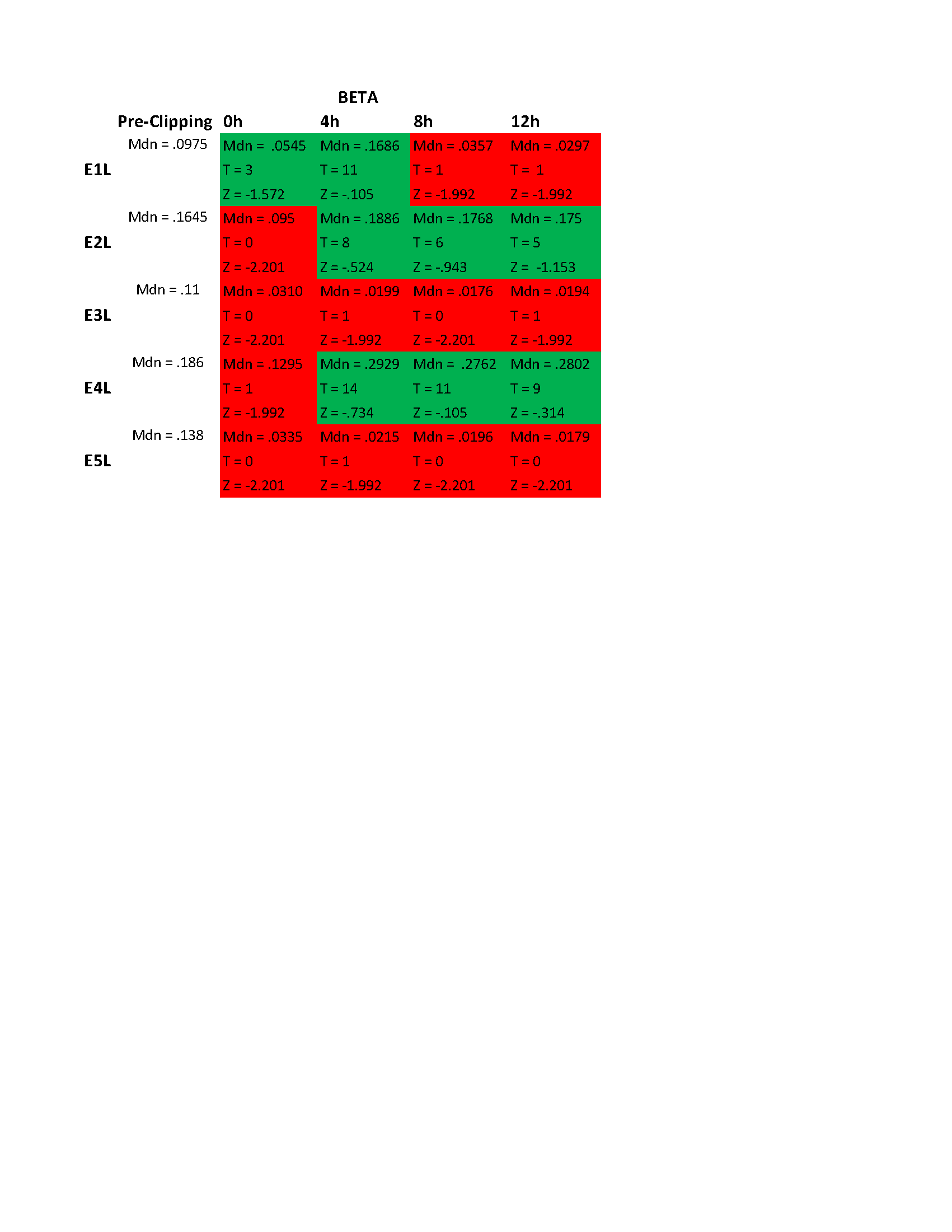

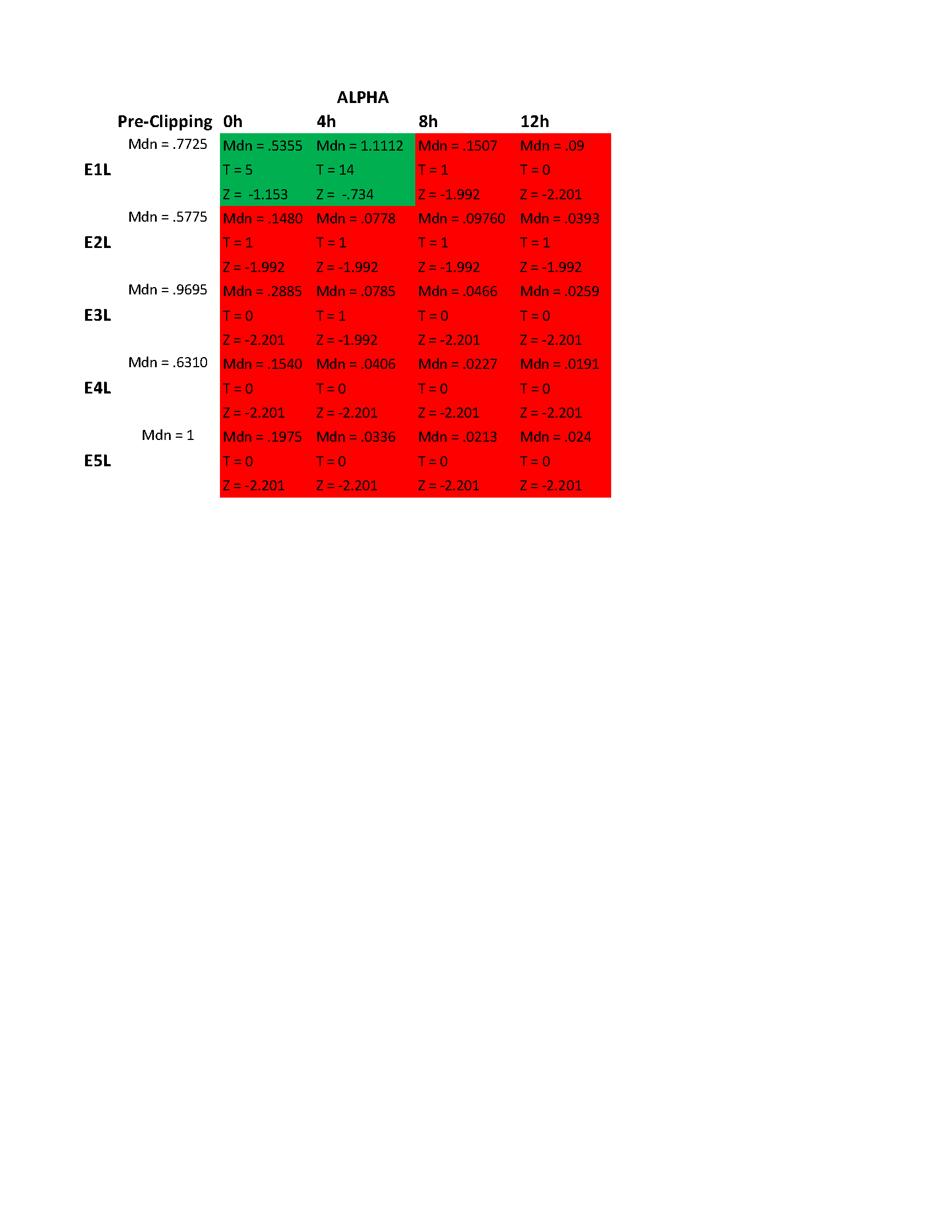


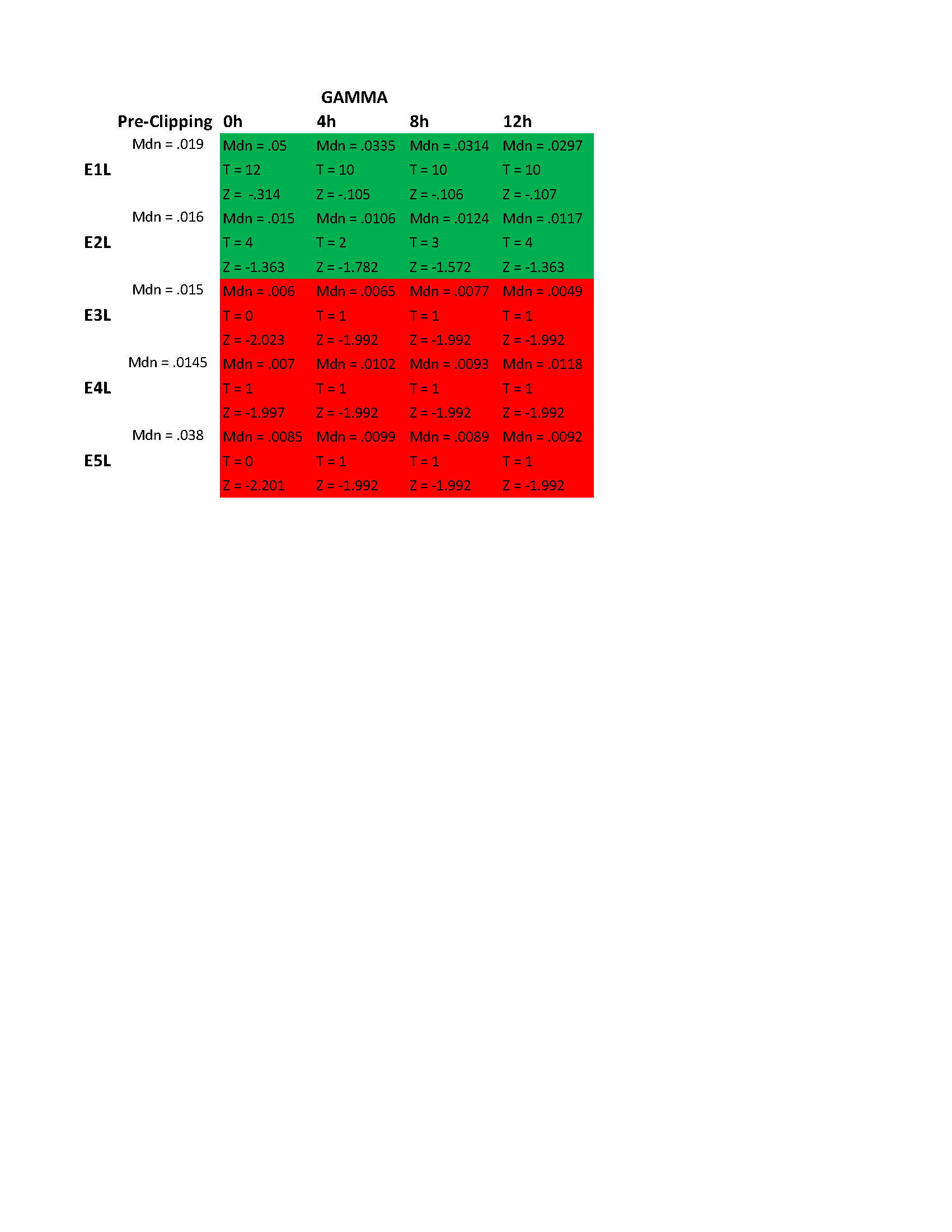


Supplementary Table 2. Statistical analysis of the spectral power of the frequency bands in the left electrodes after MCAo. The Wilcoxon matched-pairs signed-rank test was applied to observe how the spectral power of the frequency bands change 5 min after MCAo (0 h), at 4, 8, and 12 h, using the frequency bands collected 5 min prior to the MCAo as a baseline. The statistically significant drop in the spectral power of the frequency bands is represented as a red box (≤ 0.05). A green box represents no modification in the spectral power of the frequency bands (>0.05). Mdn, median; T, t-test statistic; z, z score.


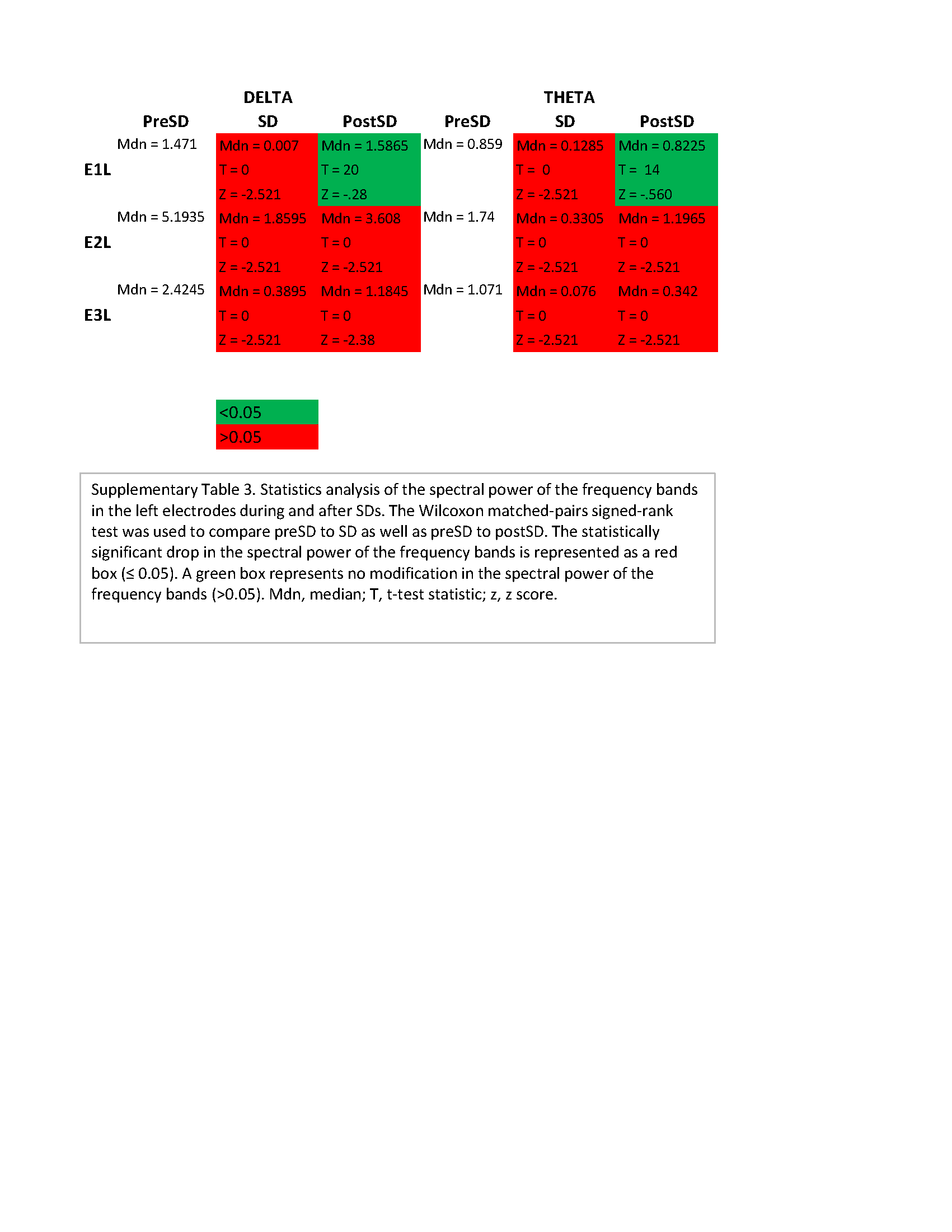


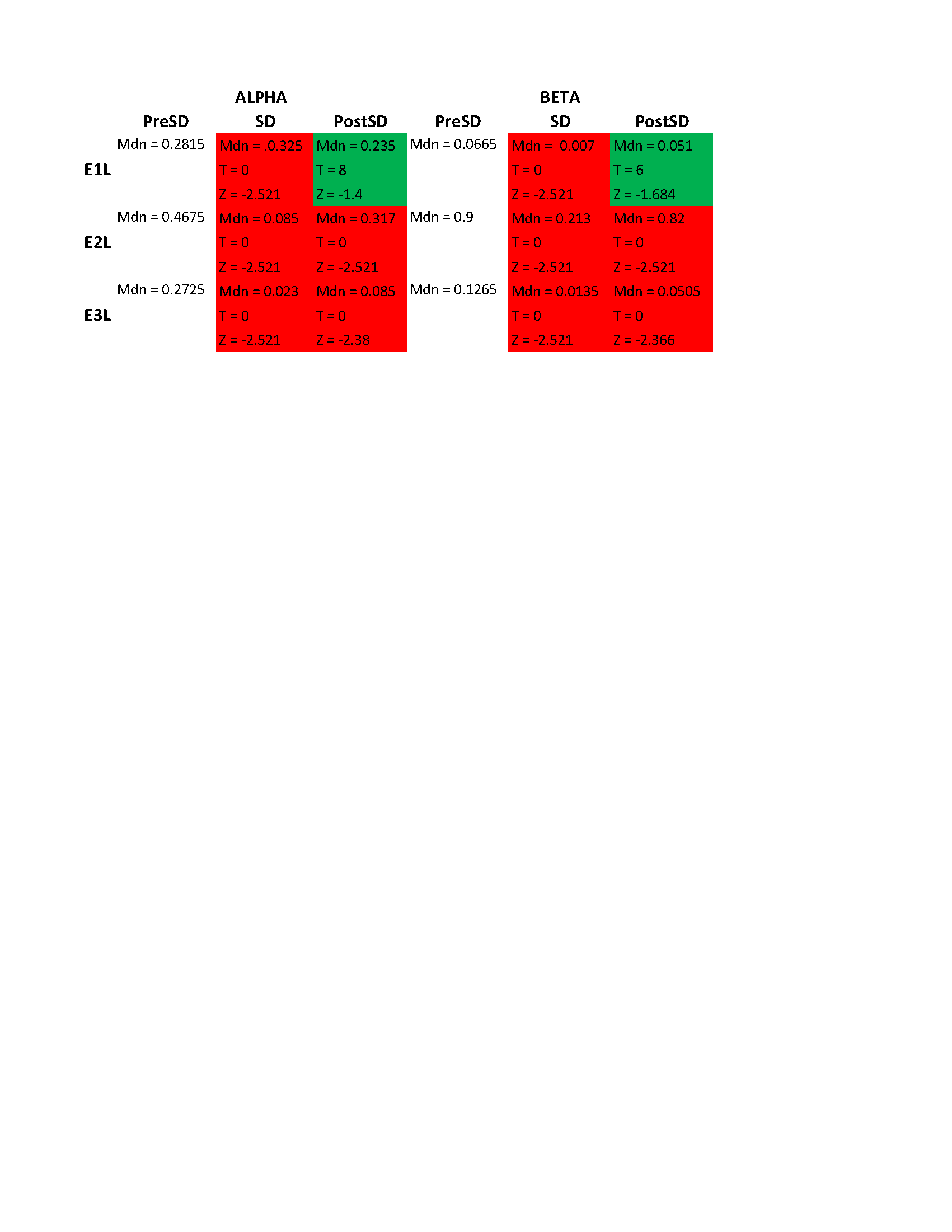


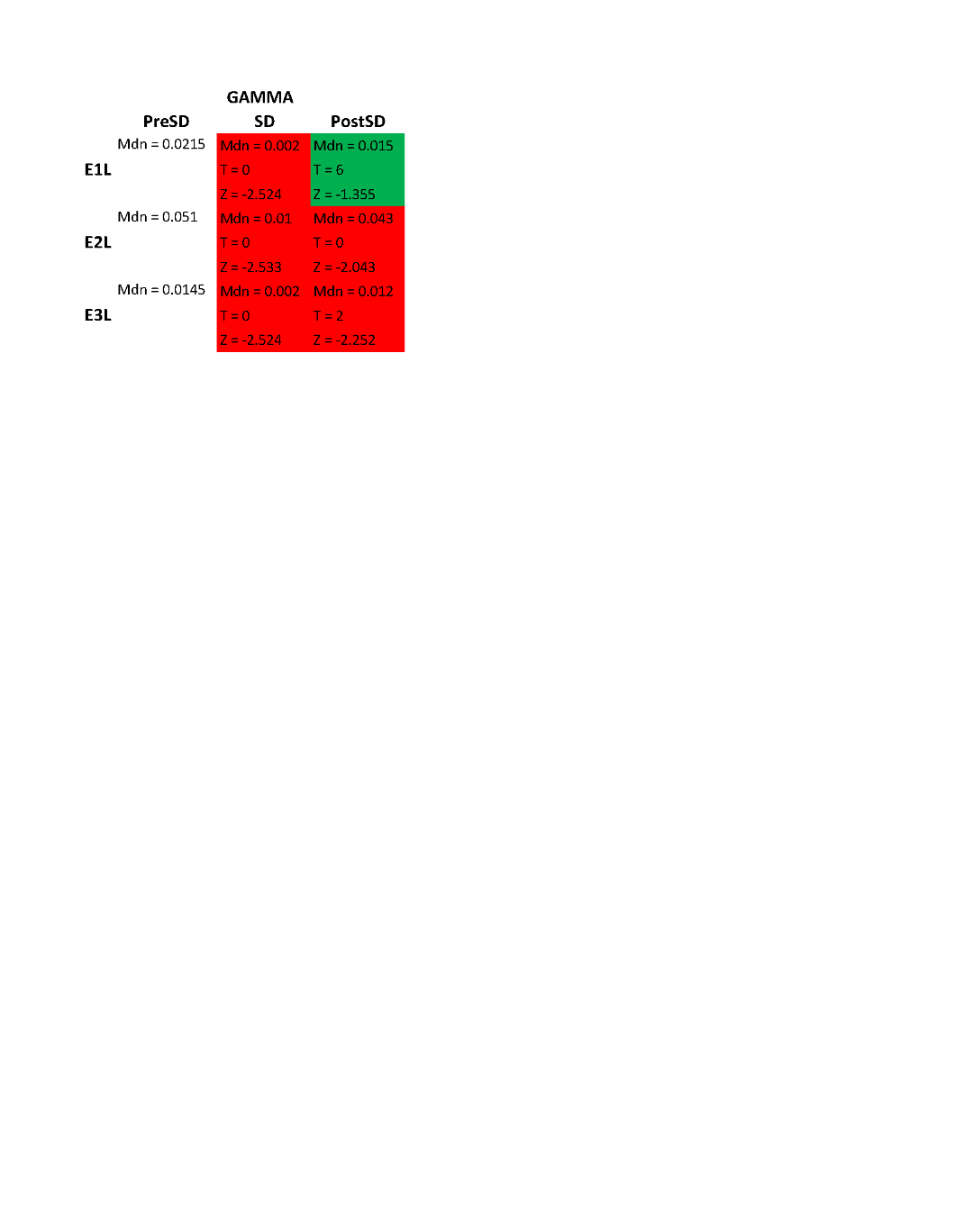


Supplementary Table 3. Statistics analysis of the spectral power of the frequency bands in the left electrodes during and after SDs. The Wilcoxon matched-pairs signed-rank test was used to compare preSD to SD as well as preSD to postSD. The statistically significant drop in the spectral power of the frequency bands is represented as a red box (≤ 0.05). A green box represents no modification in the spectral power of the frequency bands (>0.05). Mdn, median; T, t-test statistic; z, z score.
